# Comparison of Subjective Responses of Low Back Pain Patients and Asymptomatic Controls to Use of Spinal Exoskeleton during Simple Load Lifting Tasks: A Pilot Study

**DOI:** 10.3390/ijerph18010161

**Published:** 2020-12-28

**Authors:** Žiga Kozinc, Jan Babič, Nejc Šarabon

**Affiliations:** 1 Faculty of Health Sciences, University of Primorska, Polje 42, SI-6310 Izola, Slovenia; ziga.kozinc@fvz.upr.si; 2 Andrej Marušič Institute, University of Primorska, Muzejski trg 2, SI-6000 Koper, Slovenia; 3 Laboratory for Neuromechanics and Biorobotics, Jožef Stefan Institute, Jamova cesta 39, SI-1000 Ljubljana, Slovenia; jan.babic@ijs.si; 4 InnoRenew CoE, Human Health Department, Livade 6, SI-6310 Izola, Slovenia; 5 S2P, Science to Practice, Ltd., Laboratory for Motor Control and Motor Behavior, Tehnološki Park 19, SI-1000 Ljubljana, Slovenia

**Keywords:** exosuit, wearable robot, robotics, pain, occupation, ergonomics

## Abstract

Spinal exoskeletons have been suggested as an approach for the prevention and rehabilitation of occupational low back pain (LBP). While the state-of-the-art exoskeletons were shown to substantially unload the back, user acceptance is still limited. Perceived discomfort and restriction of freedom of movement are commonly reported. In this pilot study, we explored the differences in subjective responses and user impressions to using passive spinal exoskeleton during a set of simple lifting tasks between LBP patients (*n* = 12) and asymptomatic individuals (*n* = 10). Visual analog scales (0–10) were used for all assessments. Overall, the results showed mostly similar responses or slightly more positive responses to the exoskeleton from LBP patients. Most notably, the LBP patients reported a statistically significant (*p* = 0.048) higher willingness to use the device daily (5.36 ± 4.05) compared to the control group (2.00 ± 1.85) and also gave the device a higher overall grade (6.58 ± 1.98 vs. 4.30 ± 2.26; *p* = 0.021). This study has demonstrated that individuals with current LBP responded more favorably to the use of the spinal exoskeleton for simple lifting tasks. This implies that current exoskeletons could be appropriate for LBP rehabilitation, but not preventions, as pain-free individuals are less willing to use such devices. Future studies should explore whether different exoskeleton designs could be more appropriate for people with no LBP issues.

## 1. Introduction

Low back pain (LBP) remains a major worldwide issue [1]. Recently, spinal exoskeletons emerged as a possible approach to the prevention and rehabilitation of LBP in occupational environments that involve heavy-load handling and sustained static postures [2,3,4,5]. The basic mechanism by which exoskeletons are purported to help is by their provision of external torque, thereby reducing the required force exerted by the muscles and consequently also reducing the load on the spine and other joints [2,3]. Several spinal exoskeletons have recently been developed and tested. Indeed, most experiments conducted with such devices show significant benefits, such as reduced spinal compression forces and muscle activity during lifting or static bending tasks [6,7,8], as well as reduced metabolic costs of lifting [9,10]. The literature is consistent in this regard and there is little doubt that exoskeletons can significantly unload the human body. However, before workers can benefit from the exoskeletons, they must accept using them.

It has been suggested that user-device interaction is one of the most important barriers that prevent the widespread use of spinal exoskeletons [11]. Indeed, it is not uncommon that participants report at least some level of discomfort due to the exoskeleton use [12,13]. Moreover, since most workplaces do not involve solely lifting tasks or work in static postures, the exoskeletons should also be functional during other movements that do not require support (e.g., walking, turning, stair climbing, ladder climbing, etc.). The researchers have become aware of this, and consequently, the evaluation of functional performance and subjective responses during exoskeleton use has also been done [5,12,13,14]. Studies generally show no or small hindrance to functional performance induced by the current state-of-the-art spinal exoskeletons [5,13,14]. However, a major issue that remains mostly unresolved, is the subjective perception and acceptance of the device by the users, for which the abovementioned studies have shown mixed and inconclusive results. This issue has been lately addressed with qualitative methods, such as focus groups [15,16]. Although some concerns were expressed, LBP patients acknowledged the potential of the exoskeleton and generally show a willingness to use it [15,16]. While this advocates the use of exoskeletons for rehabilitation and vocational reintegration, aversion to use the exoskeleton for prevention of LBP could be expected by asymptomatic individuals.

While the aim of the exoskeleton development is to provide devices that could be used for both prevention or rehabilitation of LBP [4], the context of their use could influence the subjective responses and willingness to use the exoskeletons. Luggage handlers in a focus group reported that they would be willing to use an exoskeleton for rehabilitation, but not prevention purposes [15]. Based on the current evidence, it could be assumed that LBP patients would be willing to use the exoskeletons [5,14,15], while less is known about the attitudes of asymptomatic individuals. If the differences exist, the exoskeleton developers should consider different models for prevention and rehabilitation purposes. However, the subjective responses to exoskeleton use have never been directly compared between LBP patients and asymptomatic controls. Therefore, the purpose of this study was to assess the subjective responses and user impressions in a group of LBP patients and a group of asymptomatic individuals. Both groups performed simple lifting tasks with and without a passive spinal exoskeleton. We hypothesized that LBP patients will report higher perceived positive effects of the exoskeleton and more favorable user impression scores.

## 2. Materials and Methods

### 2.1. Participants and Study Design

For this study, 12 LBP patients (6 females, 6 males; age: 43.3 ± 7.6 years; body height = 172.7 ± 10.6 cm; body mass = 81.1 ± 17.5 kg) and 10 asymptomatic controls (5 females, 5 males; age: 39.2 ± 8.8 years; body height = 171.2 ± 10.7 cm; body mass = 71.1 ± 15.6 kg) were recruited. LBP patients were recruited through Slovenian regional healthcare centers. Inclusion criteria for the LBP group were the presence of LBP at the time of testing, with a self-rated level of more or equal to 2 on a 0–10 scale for the last 7 days. The self-reported pain score was provided by the patients 1–3 days before the testing via phone or e-mail. Inclusion criteria for both groups were that they performed heavy load handling as part of their occupation. Exclusion criteria for the control group were either the presence of current LBP or a history of LBP (defined as no LBP episodes in the last 5 years and no lifetime history of physician visits due to LBP). Exclusion criteria for both groups were pregnancy or the presence of any other musculoskeletal injuries or pain syndromes. Participants were required to sign informed consent prior to the beginning of the protocol. All measurements were conducted according to the Declaration of Helsinki and were approved by the Republic of Slovenia National Medical Ethics Committee (Approval number: 0120-199/2016-2).

### 2.2. The Exoskeleton

**Figure 1:**
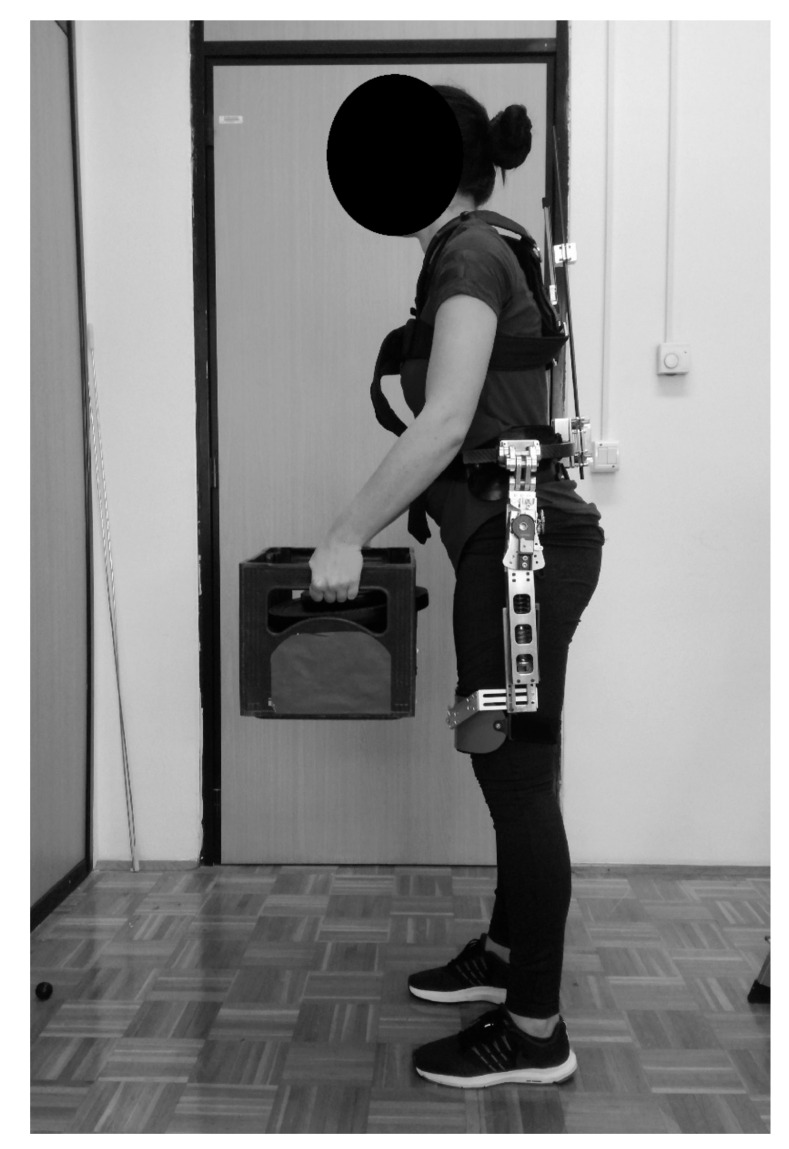
SPEXOR passive spinal exoskeleton, as used during a lifting task.

We used the passive SPEXOR exoskeleton (Figure 1), which was previously described elsewhere [3,17]. Briefly, the SPEXOR is designed to reduce loading of the low back load by generating torque with passive springs—one at each hip actuator and a bundle of three that form an elastic spinal module. Within the latter, we used 3 carbon fiber beams with 4.7 mm diameter, with the beams delivering a torque of about 16 Nm each when the user is flexed maximally (lumbar flexion = 60°). Therefore, a maximal lumbar extension torque of 48 Nm could be reached at a lumbar flexion angle of ~60°. The exoskeleton is adjustable for a wide range of body heights, which is achieved by a built-in adjustable slider at the spinal module. The important novelty of the SPEXOR exoskeleton is the inclusion of multiple self-aligning mechanisms [3] that prevent movement obstructions and discomfort.

### 2.3. Procedures and Outcome Measures

**Figure 2:**
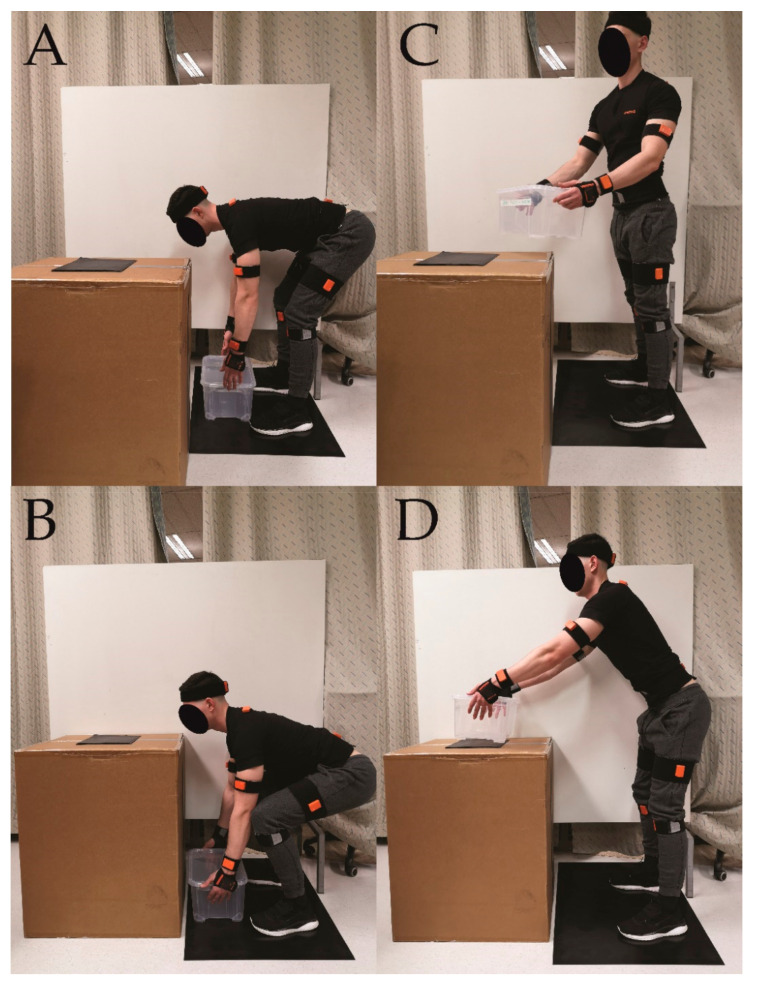
Snapshots of lifting tasks. Note that the sensors installed on the participant are unrelated to this study. The bottom position (start of the lift) differed substantially between stoop (**A**) and squat (**B**) lift. For most lifts, the participants lifted the box only upwards, with the final position in upright posture and box just in front of their hip. However, for the lifts with rotation (ROT), they had to rotate with the trunk towards the left side (**C**), while still maintaining the box close to the body. In the Free-Away lift, they had to position the box over a square placed on the table (**D**). The position from the middle of the square to the middle of the foot was 50 cm for all participants.

The participants were required to perform seven lifting tasks with and without the exoskeleton. The order of the exoskeleton conditions (either with or without), as well as the order of the tasks, were randomized among participants. All tasks involved 5 repetitions, with 5 s breaks in between. The breaks between the tasks and the conditions were set at 3 min and 15 min, respectively. All tasks were performed with a wooden 40 × 30 × 30 cm box with handles and a total mass of 10 kg (Figure 1). The tasks included free lifting, which included no specific instructions, squat lifting, and stoop lifting (participants were instructed to perform the lift predominantly by flexing at the knees (squat) or at the hips (stoop)). All tasks were performed in a ‘’normal’’ fashion (picking up and lowering a load), and with additional trunk rotation (Free_ROT, Squat_ROT, and Stoop_ROT) of 45° to the side of the non-dominant arm. The participants were given a visual target on the nearby wall to direct the load to and were instructed that the rotation of the trunk should happen throughout the lift and not only at the end of the motion. Additionally, free lifting was also performed with the instructions to move the load away (forwards) from the body as far as possible after lifting it up, before retrieving it back to the body and lowering it to the ground (Free_AWAY). This task was chosen to mirror movements like putting a load on a place away from the body (e.g., putting it on the far end of the car trunk). During the breaks, participants were encouraged to try out walking with the exoskeleton and perform a couple of sit-stands. Snapshots of the lifting tasks are available on Figure 2.

The outcome measures were all collected according to the previous studies that assessed spinal exoskeletons [5,12,13]. After each task, the participants assessed their level of local low back pain (0 = none at all; 10 = worst imaginable), the difficulty of the task (0 = very easy; 10 = very hard) and the discomfort associated with the exoskeleton (0 = none; 10 = severe; only for ‘with exoskeleton’ condition). The scores were reported on a 0–10 visual analog scale by placing a cross mark on the line that had no units depicted. In the end, the user impression questionnaire (also using visual-analog scales) was conducted that included the following questions:

- Q1: How easy is the device to put on and put off? (0 = very easy; 10 = very difficult)
- Q2: How easy is the device to adjust? (0 = very easy; 10 = very difficult)
- Q3: Are you restricted in your freedom of movement? (0 = not restricted 10 = heavily restricted)
- Q4: Does the device reduce the loading on your back? (0 = no reduction 10 = high reduction)
- Q5: Does the device interfere with the tasks you did? (0 = no interference; 10 = high interference)
- Q6: Which overall grade would you give this device? (0 = very bad; 10 = very good)
- Q7: Would you consider the device for daily use? (0 = not a chance; 10 = definitely)

### 2.4. Statistical Analysis

Statistical analysis was done in SPSS (version 25.0, SPSS Inc., Chicago, IL, USA). Descriptive statistics were calculated and reported as mean ± standard deviation and range (minimum-maximum). The differences between groups were assessed by Mann-Whitney’s U test. The effects of the exoskeleton condition (either with or without) were assessed by Wilcoxon’s signed-rank test. The level of statistical significance was set to *p* < 0.05.

## 3. Results

There were no statistically significant differences between groups regarding age (*p* = 0.177), body mass (*p* = 0.184) nor body height (*p* = 0.755). All participants successfully performed all the trials at the designated time without any complaints of major pain exacerbation. The self-reported average pain during the last week in the LBP group was 4.3 ± 1.4 (range = 2–6). The reported pain before the onset of testing was 1.98 ± 1.21 (range: 1–5) in the LBP group and 0.20 ± 0.40 (range: 0–1) in the control group.

Table 1 summarizes the results regarding local low back pain scores. The differences between the groups were statistically significant across all tasks in both conditions, except for Free_AWAY lift with the exoskeleton (*p* = 0.059). Regardless of the exoskeleton condition, the control groups reported negligible levels of local low back discomfort/pain (0.20–0.71), while the LBP groups reported mean values between 1.0 and 3.6. The exoskeleton statistically significantly reduced the pain LBP group during the Free_AWAY lift (*p* = 0.021; without exoskeleton: 2.64 ± 2.29; with exoskeleton: 1.00 ± 1.32). For the remaining lifts, no statistically significant results were obtained (*p* = 0.078–0.567; Table 1).

Task difficulty scores were very consistent across the tasks and were generally low. There was an overall trend for higher difficulty in LBP group, with statistically significant differences observed (all in the condition without exoskeleton) for Free (2.7 ± 2.9 vs. 0.5 ± 1.0; *p* = 0.032), Free_ROT (2.8 ± 2.3 vs. 0.9 ± 1.4; *p* = 0.029), Free_AWAY (1.5 ± 2.0 vs. 0.7 ± 1.2; *p* = 0.042) and Stoop (3.3 ± 2.6 vs. 0.8 ± 1.1; *p* = 0.035). There were no statistically significant effects of the exoskeleton in either group (*p* ≥ 0.392).

The discomfort scores were very similar across the tasks (range of means for overall sample = 3.4 4.2; LBP group: 2.4–3.6; control group: 4.3–4.8). The LBP groups showed lower mean discomfort values across all tasks, with statistically significant difference confirmed for Stoop (2.9 ± 1.8 vs. 4.7 ± 2.0; *p* = 0.045), and Stoop_ROT (2.4 ± 1.2 vs. 4.6 ± 1.9; *p* = 0.014).

The user impression scores are summarized in Table 2. There was a trend for the LBP group to give a more positive assessment of the exoskeleton, however, statistically, significant differences were only found for the overall score (*p* = 0.021) and the willingness for potential daily use of the device (*p* = 0.048) (see Table 2 for details).

## 4. Discussion

The purpose of this pilot study was to examine the differences between LBP patients and asymptomatic controls regarding subjective responses and user impressions related to the brief use of a passive spinal exoskeleton. The pain-reducing effect of the exoskeleton was observed in one task for the LBP group. While the results for all tasks and outcomes supported our hypothesis regarding group differences (i.e., a more favorable response from the LBP group), statistically significant differences between groups were observed for mean discomfort in 2 tasks and for two items on the user-impression questionnaire (general grade and willingness to use). This suggests that LBP patients and asymptomatic individuals perceive the exoskeletons similarly during use, however, the patients expressed a higher willingness to use it daily than their asymptomatic counterparts.

It has been stressed that user acceptance could be one of the primary culprits for the lack of widespread use of exoskeletons [11,16]. Constructing a device that would provide sufficient external supportive torque and not cause any sort of hindrance or discomfort to the user is likely impossible. State-of-the-art exoskeletons are often bulky and heavy [3,18], which is likely the cause for reported discomfort and hindrance [5,13]. Researchers have lately acknowledged such issues and started to include these aspects into consideration when conducting end-user testing [5,12,13,16]. An older study has also reported a trade-off between the amount of support and user comfort [19]. The amount of support is typically possible to adjust in state-of-the-art spinal exoskeletons [3,18], however, it is unlikely that simply reducing the level of support will substantially improve the acceptance of the device.

In this study, we used the SPEXOR exoskeletons, which have been shown to provide substantial support to the user [6,7], however, its design could be described as rather bulky and heavy [3]. Our results suggest that LBP patients respond more favorably to the use of this exoskeleton during simple lifting tasks. It could be that LBP patients saw the potential in the device for helping them with pain management and rehabilitation and were thus more likely to overlook the downsides of the device. On the other hand, asymptomatic individuals seemed to not be willing to endure this downside, with no apparent benefit. To better elucidate the reasons for these differences, focus groups should be implemented [15,16] in the future. Indeed, it has been previously stressed by focus group participants that they see a potential of the exoskeleton use in rehabilitation, but not prevention [15]. One previous study that tested the effects of the same exoskeleton reported larger positive effects on functional performance and local low back discomfort in people with a history of low-back pain, compared to the control group [14]. The authors of that study suggested that such an exoskeleton might be more applicable in secondary prevention compared to primary prevention [14]. It is beyond the scope of this paper and the authors’ knowledge to suggest how exactly exoskeleton development should be directed in the future. One possible approach would be to try to optimize the current state-of-the-art devices for patients with current LBP or other musculoskeletal issues, for whom support should be prioritized more than for asymptomatic individuals. For the latter, the developers could consider returning back to a more light-weight simplistic device, however, it should be noted that even these were reported to cause some discomfort [20,21]. With more exoskeletons available lately, a future study should compare the supportive effects and subjective responses to different models. Moreover, the expansion of subjective assessments could provide more comprehensive information. For instance, Gilardi et al. [22] used detailed questionnaires to assess workload, quality of life, and customer experience settings related to robotic technology in pediatric neurorehabilitation. In that study, the novel robotic neurorehabilitation technique was associated significant increase in the patients’ and parents’ expectations. However, physiotherapists perceived a greater workload. It would be interesting for future research to assess the expectations of LBP patients related to exoskeletons.

### Limitations

The main limitation of this pilot study is the small sample size and use of only one exoskeleton, which means that (although the used exoskeleton represents the current state-of-the-art) the results cannot be generalized to all exoskeletons. As said, future research should compare the effects of different exoskeleton models. The outcome measures were limited to subjective responses. Future studies should combine subjective assessment with biomechanical and physiological measurements. Moreover, the subjective perception was based only on putting the device on and off the body, adjustments, lifting tasks, and a short period of wearing that involved walking and sitting. A more comprehensive test battery [12] should be used to compare the responses between the groups. The load used was only moderate (10 kg). It could be that asymptomatic individuals would feel greater support if higher loads were used and would consequently recognize more potential of the exoskeleton. Finally, although the LBP patients in this group reported moderate pain levels within the last 7 days, the mean pain was relatively small just before the testing. It is possible that different responses would be observed in patients with more severe current LBP. Future studies should include larger sample sizes of LBP patients with varying pain levels, and examine subjective, physiological, and biomechanical responses while wearing different exoskeleton models.

## 5. Conclusions

This study has demonstrated that individuals with current LBP responded more favorably to the use of the spinal exoskeleton for simple lifting tasks. This implies that current exoskeletons could be appropriate for LBP rehabilitation, but not preventions, as pain-free individuals seem unwilling to use such devices. Future studies should explore whether different exoskeleton designs (e.g., simplistic light-weight exoskeletons) could be more appropriate for people without LBP issues.

## Figures and Tables

**Table 1:** Summary of local low back pain score outcomes.

Condition	Task	LBP Group				Control Group				Differences	
		Mean	SD	Min	Max	Mean	SD	Min	Max	Z	*p*
Without exo	Free	2.63	2.58	0.00	7.70	0.39	0.72	0.00	2.10	−2.580	0.010
	Free_ROT	2.87	2.20	0.00	7.10	0.39	0.73	0.00	2.30	−3.095	0.002
	Free_AWAY	2.64	2.29	0.00	7.90	0.31	0.69	0.00	2.30	−3.141	0.002
	Squat	2.23	2.60	0.00	7.90	0.31	0.88	0.00	2.80	−2.935	0.003
	Squat_ROT	2.04	2.58	0.00	7.60	0.45	0.95	0.00	2.80	−2.327	0.020
	Stoop	2.79	2.21	0.00	6.10	0.63	1.25	0.00	4.10	−2.512	0.012
	Stoop_ROT	3.49	2.94	0.00	7.50	0.70	1.30	0.00	3.90	−2.552	0.011
With exo	Free	2.11	2.01	0.00	5.20	0.21	0.63	0.00	2.10	−3.306	0.001
	Free_ROT	2.31	2.49	0.00	6.80	0.26	0.75	0.00	2.50	−2.612	0.009
	Free_AWAY	1.00	1.32	0.00	4.00	0.30	0.95	0.00	3.00	−1.885	0.059
	Squat	1.97	2.16	0.20	6.30	0.20	0.63	0.00	2.00	−3.409	0.001
	Squat_ROT	2.55	2.56	0.00	7.30	0.30	0.62	0.00	2.00	−2.945	0.003
	Stoop	2.62	2.58	0.00	8.10	0.71	1.31	0.00	4.00	−2.332	0.020
	Stoop_ROT	2.62	2.35	0.00	7.10	0.49	0.95	0.00	2.80	−2.591	0.010

ROT—rotation (lifts performed with 45° side rotation); AWAY—lifts performed with putting the box further away from the body; LBP—low back pain.

**Table 2:** Summary of user impression results.

Question Topic	LBP Group				Control Group				Differences	
	Mean	SD	Min	Max	Mean	SD	Min	Max	Z	*p*
Q1: Dooing/Doofing	4.22	2.46	0.00	8.90	6.53	3.28	0.00	7.70	−1.49	0.144
Q2: Adjusting	4.12	2.66	0.00	9.00	5.48	2.51	1.80	8.20	−1.35	0.158
Q3: Freedom to move	4.18	2.91	0.00	10.00	5.11	1.90	2.70	7.50	−0.97	0.331
Q4: Loading reduction	4.61	3.25	0.00	10.00	3.23	2.50	0.10	8.10	−0.92	0.362
Q5: Interference	2.63	2.54	0.00	10.00	3.98	2.62	0.90	8.10	−1.06	0.291
Q6: Overall grade *	6.58	1.98	3.00	10.00	4.30	2.26	1.00	8.00	−2.25	0.021
Q7: Daily Use *	5.36	4.05	0.00	10.00	2.00	1.85	0.00	5.20	−1.97	0.048

* higher scores favor the exoskeleton for Q6 and Q7; LBP: low back pain.

## Data Availability

The data presented in this study are available on request from the corresponding author. The data are not publicly available due to strict General Data Protection Regulation related policies of the authors institutions.
